# Functional Divergence of *adcyap1b* Splice Variants in Regulating Pituitary Hormone Expression in the Chinese Tongue Sole (*Cynoglossus semilaevis*)

**DOI:** 10.3390/ijms27031225

**Published:** 2026-01-26

**Authors:** Qian Zhang, Xihong Li, Yue Zhang, Wenjie Li, Zhenyu Cai, Wenteng Xu, Songlin Chen, Na Wang

**Affiliations:** 1 State Key Laboratory of Mariculture Biobreeding and Sustainable Goods, Yellow Sea Fisheries Research Institute, Chinese Academy of Fishery Sciences, Qingdao 266071, China; q2711149584@163.com (Q.Z.); lixh@ysfri.ac.cn (X.L.); zy17664080507@163.com (Y.Z.); l3174190185@163.com (W.L.); zhenyucai2001@163.com (Z.C.); xuwt@ysfri.ac.cn (W.X.); chensl@ysfri.ac.cn (S.C.); 2 Graduate School of Chinese Academy of Agricultural Sciences, Beijing 100081, China; 3 Laboratory for Marine Fisheries Science and Food Production Processes, Qingdao Marine Science and Technology Center, Qingdao 266237, China

**Keywords:** Chinese tongue sole (*Cynoglossus semilaevis*), aqauculture, sexual size dimorphism, *adcyap1*/*vip*/*ghrh*, pituitary hormones, growth hormone, alternative splicing

## Abstract

Sexual size dimorphism (SSD) refers to the phenomenon where males and females of the same species exhibit differences in overall or partial body size, and it is widespread among mammals, birds, reptiles, and fish. Notably, this dimorphism is significantly influenced by the sexually dimorphic secretion of growth hormone (*gh*), a key pituitary-derived growth regulator. Commonly, the secretion of *gh* is positively regulated by glucagon family members such as growth hormone-releasing hormone (*ghrh*) and adenylate cyclase-activating polypeptide 1 (*adcyap1*). To explore the stimulators for pituitary hormones (especially *gh*) in the teleost, we performed genome-wide identification and functional characterization of the glucagon family on Chinese tongue sole (*Cynoglossus semilaevis*) that exhibits typical female-biased sexual size dimorphism. Four members of *adcyap1*/vasoactive intestinal polypeptide(*vip*)/*ghrh* family and ten members of their receptor family were identified. Expression pattern analysis revealed high expression of adenylate cyclase-activating polypeptide 1b (*adcyap1b*) and its receptors in the brain. Moreover, two alternative splice variants for the *adcyap1b* gene were discovered, resulting from the skipping of exon 4. Following the acquisition of the two eukaryotic recombinant protein splice variants (ADCYAP1b_tv1 and ADCYAP_tv2) from HEK 293T cells, incubation experiments were conducted using *C. semilaevis* pituitary cell line. The results demonstrated that both variants promoted the expression of *gh*, pro-opiomelanocortin (*pomc*), and corticoliberin (*crh*), but ADCYAP1b_tv1 had a significantly stronger effect and uniquely stimulated prolactin (*prl*) and somatolactin (*sl*). This study demonstrates a functional divergence between the two ADCYAP1b splice variants in teleosts, with ADCYAP1b_tv1 acting as a more potent and versatile pituitary hormone stimulator. Further research on their receptor-binding affinity and downstream signaling pathways would be valuable for exploring the mechanism underlying sexual size dimorphism.

## 1. Introduction

Sexual size dimorphism, the difference in body size between sexes, occurs across various species including mammals, birds, reptiles, and fish [1,2]. The secretion of *growth hormone* (*gh*) from the pituitary is a pivotal regulator of organismal growth [3] and itself frequently displays sexual dimorphism. Sexually dimorphic expression of *gh* gene drives sex-specific differences in growth, development, and physiological functions, thereby contributing to sexual growth dimorphism [4].

Understanding the regulation of this differential *gh* secretion is essential. The conventional view holds that in mammals and birds, it is primarily under the positive control of growth hormone-releasing hormone (GHRH), a glucagon family member [5,6]. However, in fish models such as goldfish (*Carassius auratus*) and medaka (*Oryzias latipes*), the positive regulation of *gh* is primarily mediated by pituitary adenylate cyclase-activating polypeptide (PACAP) encoded by the adcyap1 gene—another member of the glucagon family [7,8,9]. PACAP was first isolated from the cattle hypothalamus [10,11] and influenced the secretion of multiple hormones within both the hypothalamic–pituitary–gonadal (HPG) and hypothalamic–pituitary–adrenal (HPA) axes by activating the ERK and cAMP/PKA signaling pathways [12,13,14].

The Chinese tongue sole (*Cynoglossus semilaevis*) is a flatfish endemic to China and is known for its pronounced female-biased growth dimorphism [15]. Although previous studies have begun to elucidate the mechanisms behind this phenomenon [16,17,18,19], the sexual dimorphic regulation of *gh* secretion remains a key unresolved question. Recently, using single-cell multi-omics analysis, we found that *adcyap1*-positive GABAergic neurons are 2.32 times more abundant in females than in males (unpublished data). It remains to be investigated whether *adcyap1* serves as a central regulatory function in pituitary hormone secretion, rather than *ghrh*.

To address this issue, this study firstly conducted a genome-wide identification and evolutionary analysis of the glucagon family genes (*ghrh*/*adcyap1*/*vip*) and their receptors in the Chinese tongue sole. Furthermore, we examined the expression patterns of ligands and their receptors in key growth-axis tissues across different sexes. Finally, the functional role of ADCYAP1b in regulating pituitary hormone secretion was elucidated through in vitro incubation assays. This work provides a crucial foundation for understanding the regulatory mechanisms behind pituitary hormone secretion.

## 2. Results

### 2.1. Genome-Wide Identification and Evolutionary Analysis of Members of the Glucagon Family and Its Receptors in C. semilaevis

**Figure 1:**
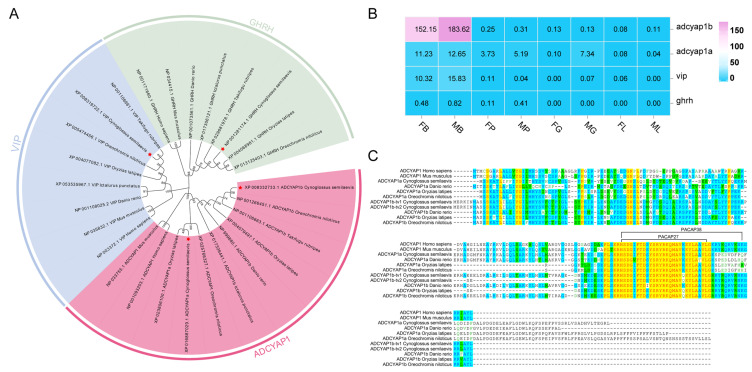
The evolutionary tree of ADCYAP1/GHRH/VIP and its expression patterns in *C. semilaevis*. (**A**) Phylogeny of ADCYAP1/GHRH/VIP (neighbor-joining method, MEGA 11.0) from *C. semilaevis*, *O. latipes*, *O. niloticus*, *T. rubripes*, *D. rerio*, *I. punctatus*, *H. sapiens*, and *M. musculus*. *C. semilaevis* proteins were marked with red stars. (**B**) Heatmap of *adcyap1a*/*1b*, *ghrh*, and *vip* in female/male tissues. FB: female brain; MB: male brain; FP: female pituitary; MP: male pituitary; FG: female gonad; MG: male gonad; FL: female liver; and ML: male liver. Expression levels were quantified as FPKM based on two-year-old *C. semilaevis* RNA-Seq data. (**C**) ADCYAP1 protein alignment (*H. sapiens*, *M. musculus*, *C. semilaevis*, *D. rerio*, *O. latipes*, and *O. niloticus*).

By genome-wide screen, four glucagon family members were identified from *C. semilaevis* (Table 1). A phylogenetic tree of glucagon family genes was constructed by aligning homologous protein sequences in *C. semilaevis*, *Oryzias latipes*, *Oreochromis niloticus*, *Takifugu rubripes*, *Danio rerio*, *Ictalurus punctatus*, *Homo sapiens,* and *Mus musculus*. The results (Figure 1A) show that the sequences are primarily clustered into the following three major branches: ADCYAP1, VIP, and GHRH. Furthermore, ADCYAP1 branch is subdivided into the ADCYAP1B and ADCYAP1A subclades. *C. semilaevis* ADCYAP1A and ADCYAP1B are both closely related with ADCYAP1B of *Oreochromis niloticus* and *Oryzias latipes*. In VIP branch, *C. semilaevis vip* clusters closely with the VIP sequences of *T. rubripes* and *O. latipes*. In GHRH branch, *C. semilaevis* GHRH clusters closely with the GHRH sequences of *O. latipes* and *O. niloticus*. In addition, the evolutionary tree clearly illustrates the phylogenetic patterns of the ADCYAP1, VIP, and GHRH families in teleost fishes and mammals.

By using our previous data of two-year-old *C. semilaevis* tissues RNA-seq, *adcyap1b* showed the highest expression level in the brains, compared with the other members of glucagon family members (Figure 1B). Noticeably, the expression of *ghrh* was barely detectable in the brain. By comparing the ADCYAP1 protein sequences of teleosts and mammals, it was found that most proteins have conserved sequences near the C-terminus. Among them, ADCYAP1b is more like mammalian PACAP38, while PACAP27 shows higher similarity between fish ADCYAP1a and mammalian ADCYAP1 proteins (Figure 1C).

**Figure 2:**
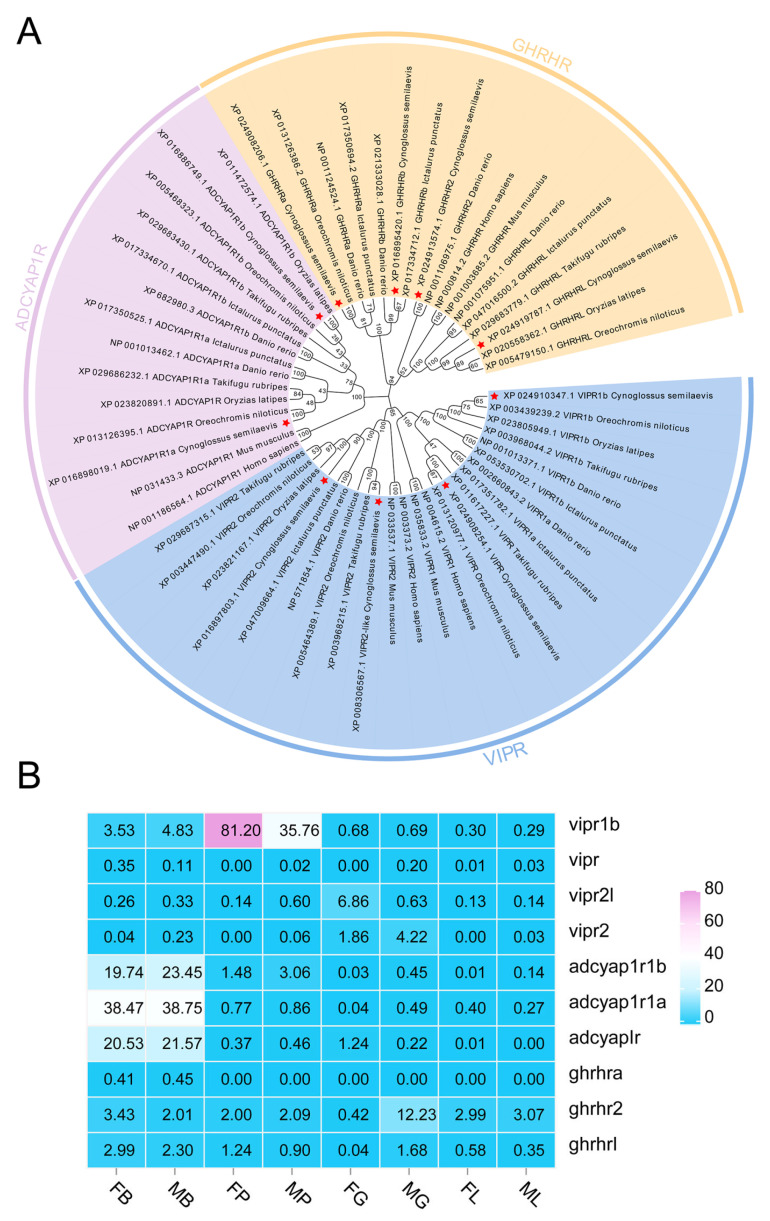
Evolutionary tree and tissue expression of ADCYAP1R/GHRHR/VIPR in *C. semilaevis*. (**A**) Phylogenetic tree of ADCYAP1R/VIPR/GHRHR (neighbor-joining method, MEGA 11.0) from *C. semilaevis*, *O. latipes*, *O. niloticus*, *T. rubripes*, *D. rerio*, *I. punctatus*, *H. sapiens,* and *M. musculus*. Ten members from *C. semilaevis* were marked with red stars. (**B**) Heatmap of *adcyap1r*/*ghrhr*/*vipr* mRNA in female/male *C. semilaevis* tissues. Expression levels were quantified as FPKM based on two-year-old *C. semilaevis* RNA-Seq data.

To further explore ligand–receptor interaction mechanisms, the genome-wide identification for the glucagon family receptors was also conducted. As a result, ten members were screened from *C. semilaevis* and aligned with other teleosts including *O. latipes*, *O. niloticus*, *T. rubripes*, *D. rerio*, *I. punctatus*, as well as *H. sapiens* and *M. musculus*. The phylogenetic tree (Figure 2A) reveals that the sequences are clustered into the following three major branches: VIPR branch, ADCYAP1R branch, and GHRHR branch. For mammals, VIPR branch contained VIPR1 and VIPR2. In teleosts, four members including VIPR1a, VIPR1b, VIPR2, and VIPR2l are included. Similarly, teleost ADCYAP1R branch includes ADCYAP1R1a and ADCYAP1R1b subtypes, different from one member in mammals. For GHRHR branch, still one member is identified from mammals, while four members including GHRHRa, GHRHRb, GHRHR2, and GHRHRl are screened from the teleost species. The RNA-seq data of two-year-old *C. semilaevis* tissues (Figure 2B) revealed that three receptors of *adcyap1r* showed a relatively high expression level in the brains. In the pituitary gland, *vipr1b* exhibited the highest abundance.

### 2.2. Expression Patterns and Alternative Splicing Analysis of the adcyap1 Gene in C. semilaevis

**Figure 3:**
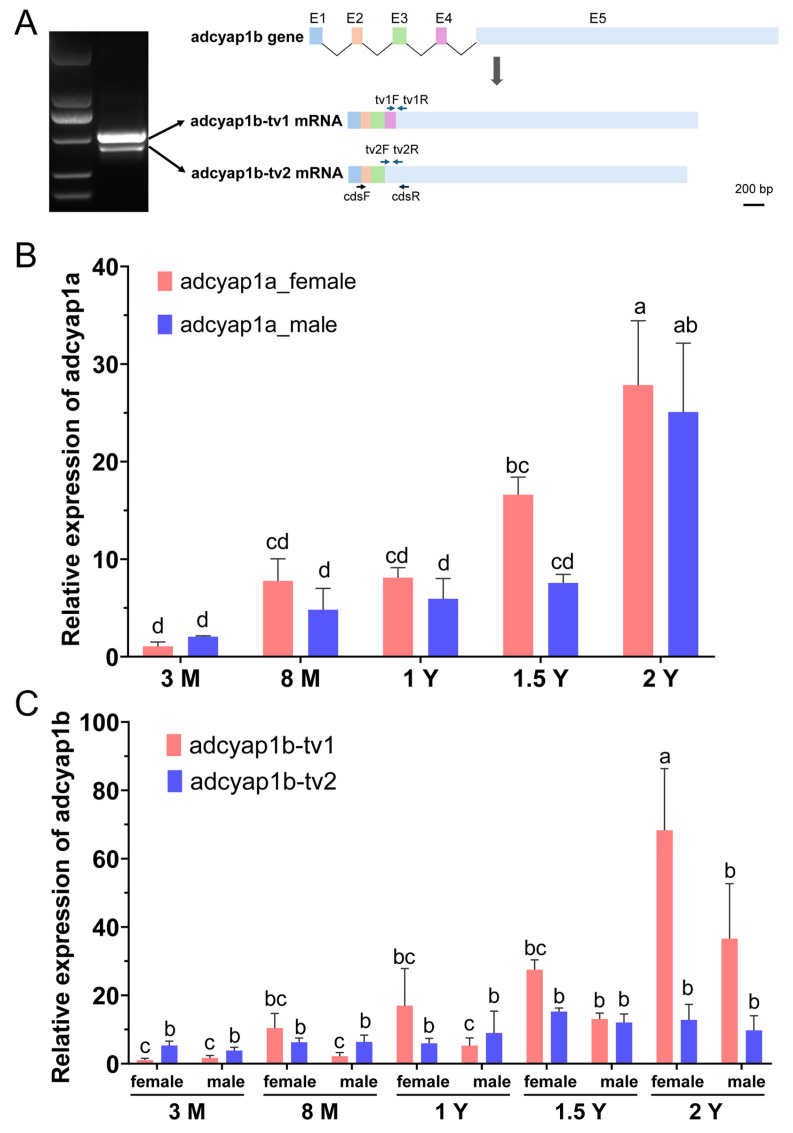
*Adcyap1* expression in *C. semilaevis* brain (developmental stages) and *adcyap1b* alternative splicing. (**A**) *adcyap1b* produces two variants (*adcyap1b_tv1*/*tv2*) via alternative splicing. RT-PCR agarose gel shows specific bands (primers cdsF/cdsR); *adcyap1b* genomic structure are illustrated and five exons were represented as E1, E2, E3, E4, and E5 (scale bar = 200 bp). Primers tv1F/tv1R and tv2F/tv2R are separately designed for qPCR assays of *adcyap1b_tv1* and *adcyap1b_tv2*. (**B**) *adcyap1a* relative expression in female/male brains (3 M, 8 M, 1 Y, 1.5 Y, and 2 Y; qPCR). (**C**) *adcyap1b_tv1*/*tv2* relative expression in female/male brains (above stages; qPCR). Group differences (*p* < 0.05, different letters; LSD test).

During the verification process of the *adcyap1b* gene sequence, two specific PCR bands were amplified by primers cdsF and cdsR (Figure 3A). Sequencing analysis revealed that the sizes of these two bands were 546 bp and 441 bp, respectively, with the difference primarily resulting from exon 4 skipping. This indicates that the *adcyap1b* gene of *C. semilaevis* undergoes alternative splicing, and the gene can be transcribed into two alternative splice variants, *adcyap1b_tv1* and *adcyap1b_tv2*.

The relative expression levels of the genes were calculated using the 2^−∆∆Ct^ method. The results showed that the expression of the *adcyap1a* gene exhibited a gradual upregulation trend with increasing age, reaching a common peak in both female and male individuals at 2 years of age (2 Y). The *adcyap1b* gene displayed differential expression through its distinct transcript variants (*adcyap1b_tv1* and *adcyap1b_tv2*), among which the expression dynamics of *adcyap1b_tv1* were more specific.

### 2.3. Recombinant Eukaryotic Expression and Functional Analysis of Two Splice Variants of ADCYAP1b

**Figure 4:**
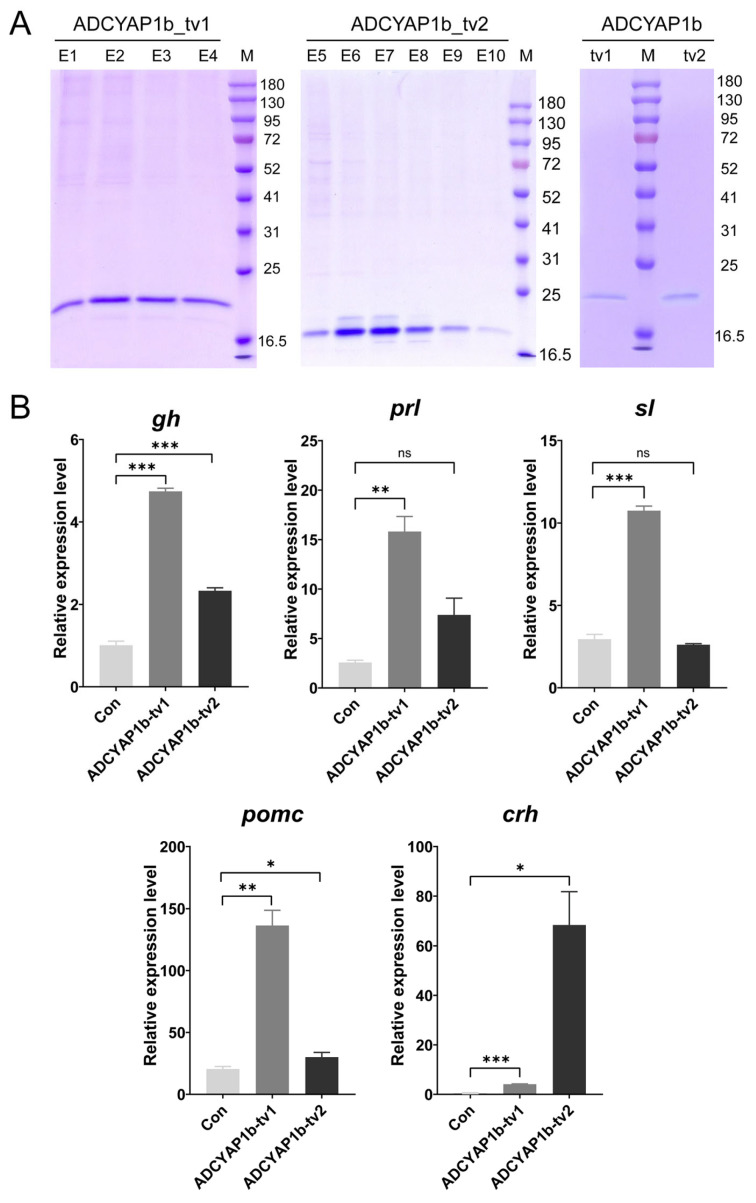
ADCYAP1b_tv1/2 eukaryotic recombinant protein expression and effects on pituitary hormones. (**A**) Intracellular samples and culture supernatants collected for purification following the transfection of pcDNA-ADCYAP1b_tv1/tv2 into HEK 293T cells; E1–E4: ADCYAP1b_tv1 gradient elution fractions; E5–E10: ADCYAP1b_tv2 fractions. SDS-PAGE shows purified proteins; M: prestained protein marker. (**B**) Relative expression of *gh*, *prl*, *sl*, *pomc*, and *crh* in pituitary cells after treatment with recombinant proteins. * *p* < 0.05, ** *p* < 0.01, and *** *p* < 0.001 (vs. control); “ns”: no significant difference.

Two splice variants of ADCYAP1b (ADCYAP1b_tv1 and ADCYAP1b_tv2) were successfully expressed in a recombinant eukaryotic system, exhibiting the following distinct subcellular localization patterns: ADCYAP1b_tv1 was expressed as an intracellular protein, while ADCYAP1b_tv2 was secreted into the cell culture supernatant. Both proteins were purified by His-tag affinity chromatography and eluted with an imidazole gradient. Approximately 200 µg of each purified protein was obtained, desalted to remove imidazole, and stored in DMEM for subsequent analysis (Figure 4A).

Following in vitro incubation assay using pituitary cells, the quantitative real-time PCR (qRT-PCR) analysis based on the 2^−∆∆Ct^ method was conducted to assess the regulatory effects of ADCYAP1b_tv1 and ADCYAP1b_tv2 proteins on the expression of growth- and endocrine-related genes. Results demonstrated that both variants significantly promoted the transcriptional levels of *gh*, *pro-opiomelanocortin* (*pomc*), and *corticoliberin* (*crh*) compared to the control group (*p* < 0.05), with ADCYAP1b_tv1 exerting a notably stronger promotional effect on *gh* and *pomc* compared to ADCYAP1b_tv2. In contrast, a significant promotional effect on the *prolactin* (*prl*) and *somatolactin* (*sl*) genes was only observed in the recombinant ADCYAP1b_tv1 treatment group.

Combined with the expression patterns of two splice variants, the female-biased expression of *adcyap1b_tv1* in the brain is implicated as a critical driver of sexual size dimorphism, likely through stimulating greater *gh* production in the female pituitary gland.

## 3. Discussion

Research on the regulation of *gh* secretion is crucial for understanding sexual size dimorphism (SSD) across species. In mammals, existing evidence indicates that *gh* secretion is regulated not only by classical *ghrh* signaling but is also influenced by other factors such as leptin and ghrelin [20,21,22]. In this study, female-biased SSD species *C. semilaevis* was used to investigate the regulatory mechanisms of *gh*.

Similar with other teleosts, two *adcyap1* genes including *adcyap1a* and *adcyap1b* were identified from *C. semilaevis*. By comparison with the ADCYAP1 protein, teleost ADCYAP1b exhibited more similarity with mammalian PACAP38. It implied that *adcyap1a* gene may have originated from a fish-specific genome duplication event [23]. The *adcyap1* gene is widely expressed in mammals, with a distribution that spans the central and peripheral nervous systems as well as non-neural tissues ranging from the adrenal glands and gonads to the pancreas (reviewed by [24]). In *C. semilaevis*, *adcyap1b* shows high expression exclusively in brain tissues, while *adcyap1a* exhibits moderate expression in brains, pituitary glands, and male gonad. These differences in mRNA localization between the two *adcyap1* paralogs suggest their functions might have diverged concomitant with the occurrence of genome duplication.

Moreover, two alternative splicing transcripts of *C. semilaevis adcyap1b*, resulting from exon 4 skipping, were identified. In mammals, the *adcyap1* gene employs multiple promoters to generate tissue-specific transcripts for unique physiological functions [25,26]. Illustratively, it has been demonstrated that in rats, a specific *adcyap1* promoter expresses a distinct mRNA in the testis, which is crucial for maintaining the process of spermatogenesis [25,27].

Evidence from fish species including goldfish and medaka has suggested that *adcyap1* may function as a hypophysiotropic factor regulating pituitary hormone secretion [7,8,9]. In the present study, two splice proteins of ADCYAP1b in *C. semilaevis* both promote the secretion of *gh*, *pomc*, and *crh*, with ADCYAP1b_tv1 exhibiting a stronger regulatory effect. It implied that *adcyap1b* may play crucial roles in growth and development, feeding behavior, and sex reversal [28,29,30,31]. Moreover, ADCYAP1b_tv1 can also regulate osmoregulation and reproductive behavior by promoting the secretion of *prl* [32,33] and *sl* [34,35].

In addition, the effects of ADCYAP1 on pituitary hormones appear to be species-specific, as evidenced by comparison across different fish species. For instance, medaka ADCYAP1 recombinant protein does not significantly promote *gh* but significantly stimulates both *prl*, *sl,* and luteinizing hormone subunit beta (*lhb*) in both females and males [9]. Additionally, it exhibits an inhibitory effect on male *pomc*. In another fish-goldfish, ADCYAP1 analogs could stimulate the release of pituitary hormones, including *gh* and *lhb* [7].

Overall, the study establishes ADCYAP1b, particularly its tv1 variant, as a potent and multifunctional pituitary hormone regulator in *C. semilaevis*.

## 4. Materials and Methods

### 4.1. Ethics Statement

All fish were euthanized by submerging in MS-222 (120 mg/L) solution prior to the experiments. The sampling and treatment of *C. semilaevis* was approved by Institute Animal Care and Use Committee of the Yellow Sea Fisheries Research Institute, Chinese Academy of Fishery Sciences (approval number YSFRI-2025017).

### 4.2. Fish Sampling and Cell Culture

All healthy *C. semilaevis* at 3 months, 8 months, 1 year, 1.5 years, and 2 years of age were sourced from Huanghai Aquaculture Co., Ltd. (Haiyang, China). The fish were reared in a recirculating aquaculture system, fed a small amount of diet twice daily (at 8:00 a.m. and 4:00 p.m.), and maintained at a water temperature of 18–22 °C.

*C. semilaevis* pituitary cells were cultured in L-15 medium supplemented with 10% fetal bovine serum (Gibco, Grand Island, NY, USA) and maintained in an incubator at 24 °C.

### 4.3. Genome-Wide Identification and Evolutionary Analysis of the Glucagon Family and Its Receptors in C. semilaevis

The protein sequences of *adcyap1*, *vip*, and *ghrh* from *C. semilaevis* were retrieved from the NCBI database for phylogenetic analysis. Corresponding gene sequences from other teleosts and mammals, including *Oryzias latipes*, *Takifugu rubripes*, *Danio rerio*, and *Homo sapiens*, were also selected. Sequence alignment was performed using ClustalW in MEGA (Version 11.0.13), and a phylogenetic tree was constructed using the neighbor-joining method. The resulting tree was subsequently visualized and refined using the EvolView webserver (http://www.evolgenius.info/evolview/#/treeview (accessed on 1 September 2025)).

We utilized RNA-seq data of the brain, liver, gonads, and pituitary from 2-year-old fish [16] to profile the mRNA distribution in *C. semilaevis*. The relative mRNA abundances of the above-mentioned *adcyap1*/*vip*/*ghrh* ligand and receptor family member genes were obtained from these data, and a heatmap was created using the Omicshare Tool (https://www.omicshare.com/tools (accessed on 1 September 2025) to visualize the expression profiles. The relevant ADCYAP1 protein sequences from various species were aligned with Vector NTI software (Version 11.5.1) to generate a protein sequence alignment figure.

### 4.4. Expression Profiling and Alternative Splicing Analysis of the adcyap1 Gene in C. semilaevis

To examine the expression patterns of the *adcyap1b* gene across developmental stages, PCR amplification was initially conducted using cDNA from the brain tissue of 1.5-year-old *C. semilaevis*. The reaction was performed in a 50 μL system containing 25 μL KOD Mix, 1.5 μL each of coding region-specific primers (cdsF and cdsR) (Table 2), 20 μL ddH_2_O, and 2 μL cDNA template. The PCR protocol consisted of an initial denaturation at 98 °C for 10 s, followed by 40 cycles of denaturation at 98 °C for 10 s, annealing at 58–62 °C for 5 s, and extension at 68 °C for 25 s, with a final extension at 72 °C for 7 min. This process led to the identification of two alternative splice variants of *adcyap1b*, designated *adcyap1b_tv1* and *adcyap1b_tv2*.

Two pairs of quantitative PCR (qPCR) primers (tv1F/tv1R and tv2F/tv2R) (Table 2) were designed to specifically target each splice variant. qPCR was performed using cDNA derived from brain tissues of four female and four male *C. semilaevis* at different developmental stages. Each 10 µL reaction contained 5 µL SYBR Taq, 0.2 µL each of forward and reverse primers, 1 µL diluted cDNA, and 3.6 µL H_2_O. The thermal cycling conditions were as follows: 95 °C for 30 s; 40 cycles of 95 °C for 3 s; and 60 °C for 33 s, followed by a melting curve analysis (95 °C for 15 s, 60 °C for 60 s, and 95 °C for 15 s). Relative expression levels were calculated using the 2^−∆∆Ct^ method, analyzed by LSD multiple comparison tests, and visualized using GraphPad Prism 8.0.2.

### 4.5. Recombinant Eukaryotic Expression of Two ADCYAP1b Splice Variants

To functionally characterize *adcyap1b* in *C. semilaevis*, we performed recombinant eukaryotic expression and purification of its two splice variants using the HEK 293T mammalian cell line. The coding sequences of ADCYAP1b_tv1 and ADCYAP1b_tv2 were codon-optimized, and an exogenous signal peptide (MYRMQLLSCIALSLALVTNS) was added to the N-terminus, followed by a C-terminal Flag-His purification tag. The modified fragments were cloned into the pcDNA3.1(–) vector via EcoRI and HindIII restriction sites, resulting in the recombinant constructs pcDNA3.1-ADCYAP1b_tv1 and pcDNA3.1-ADCYAP1b_tv2.

These plasmids were separately transfected into HEK 293T cells using a liposome-based reagent. At 48–72 h post-transfection, cell culture supernatants and lysates were collected and analyzed by Western blot with an anti-Flag antibody to confirm recombinant protein expression. Recombinant ADCYAP1b_tv1 (intracellular) and ADCYAP1b_tv2 (secreted) were successfully purified by His-tag affinity chromatography. The purified proteins were then desalted to remove imidazole and stored in DMEM medium for subsequent functional assays.

### 4.6. Effect of ADCYAP1b Recombinant Proteins on the Pituitary Cells

To investigate the regulatory effects of the two recombinant *adcyap1b* splice variants (ADCYAP1b_tv1 and ADCYAP_tv2) on pituitary hormone secretion in *C. semilaevis*, we conducted an in vitro incubation assay using pituitary cells. The experiment utilized tenth-passage female pituitary cells cultured in the laboratory. Cells were divided into the following three groups: the ADCYAP1b_tv1 treatment group, the ADCYAP1b_tv2 treatment group, and an untreated control group maintained in normal culture medium. Each group consisted of two replicate flasks (25 cm^2^). For the treatment groups, 0.5 mL of recombinant protein (0.05 μg/μL) was administered to each flask, resulting in a total of 25 μg per flask. Following a 48 h incubation at 24 °C, total RNA was extracted using the Trizol method and reverse-transcribed into cDNA. The expression levels of pituitary genes (*gh*, *prl*, *sl*, *pomc*, and *crh*) (Table 2) were quantified by quantitative real-time PCR (qRT-PCR) with β-actin as the reference gene. Relative gene expression was calculated using the 2^−ΔΔCt^ method, and statistical analysis was performed using Student’s *t*-test in GraphPad Prism 8.0.2.

## 5. Conclusions

In conclusion, this study provides a comprehensive view of the glucagon family in *C. semilaevis*, from genome-wide identification to functional characterization. The results demonstrate that *C. semilaevis adcyap1b* gene produces two functionally distinct ligands via alternative splicing, which contributes to the regulation of pituitary hormone expression. Specifically, both variants promoted the expression of *gh*, *pomc*, and *crh*, but ADCYAP1b_tv1 had a significantly stronger effect and uniquely stimulated *prl* and *sl*. Nevertheless, the detailed molecular pathways and specific cell groups through which adcyap1b acts require further clarification. Targeted investigations combining single-cell multi-omics and molecular experiments are warranted to address these questions, thereby elucidating the detailed mechanism on sexual size dimorphism.

## Figures and Tables

**Table 1:** Sequence features of *adcyap1*/*vip*/*ghrh* ligand and receptor family members.

Name	Gene ID	Gene Length (bp)	ORF Length (bp)	Amino Length (aa)	Chr	Location	No. of Exons
*adcyap1a*	103377449	17,097	642	213	3	14843555–14860651	11
*adcyap1b*	103396432	5904	546	181	20	9316414–9322317	5
*vip*	103387011	3804	459	152	12	3323366–3327169	6
*ghrh*	103386346	5037	477	158	11	11246313–11251349	6
*adcyap1r1a*	103396405	19,932	1464	487	20	8971846–8991777	16
*adcyap1r1b*	103399891	19,190	1539	512	2	14104666–14123855	14
*vipr*	103396216	21,153	1335	444	20	6167500–6188652	15
*vipr1b*	103377222	42,437	1332	443	3	10461366–10503802	15
*vipr2*	103396749	4950	1092	363	20	13743807–13748756	9
*vipr2-like*	103377507	8238	1344	447	3	15936274–15944511	13
*ghrhrl*	103398124	13,620	1248	415	2	9758286–9771905	13
*ghrhra*	103396404	8848	1258	418,418	20	8963214–8972061	13
*ghrhrb*	103399903	32,425	1425	444,474	2	14125482–14157906	15
*ghrhr2*	103381621	34,534	1164	387	8	3652316–3686849	20

**Table 2:** Primers in present study.

Primer	Primer Sequence
cdsF	ATGGAGCGCAAGATAAACATG
cdsR	CTACAAATAGGCAAGCCGGCG
tv1F	GCATTCTCTGATGACAGTTCGT
tv1R	ACCGCAGCCAGGTATTTCT
tv2F	GAGAAGAGTGAAGACAACAGCA
tv2R	CTACAAATAGGCAAGCCG
*gh*F	ATCCACGCAGCCGGTTATAG
*gh*R	CTCATGCTTGTTGTCGGGGA
*prl*F	ACAGTGGAGGCAACGACA
*prl*R	CAACGCAGGACTTTCAGG
*sl*F	CTGGACTGTAAGGACGAGC
*sl*R	CTGAGATGCGGGAGATGA
*pomc*F	GTCCTCTGTGGCTATTGGT
*pomc*R	ATGGCACTGCTCTTGGAG
*crh*F	CCGTCCTCCTCCCTGTAT
*crh*R	GCCCTGATGTTCCCAAAT
*actin*F	TTCCAGCCTTCCTTCCTT
*actin*R	TACCTCCAGACAGCACAG

## Data Availability

The original contributions presented in this study are included in the article. Further inquiries can be directed to the corresponding author.

## Abbreviations

The following abbreviations are used in this manuscript:

*adcyap1*	adenylate cyclase-activating polypeptide 1
*adcyap1a*	adenylate cyclase-activating polypeptide 1a
*adcyap1b*	adenylate cyclase-activating polypeptide 1b
*vip*	vasoactive intestinal polypeptide
*ghrh*	growth hormone-releasing hormone
*gh*	growth hormone
*sl*	somatolactin
*prl*	prolactin
*pomc*	pro-opiomelanocortin
*crh*	corticoliberin
*lhb*	luteinizing hormone subunit beta
